# Genetic Diversity, Population Structure, and Linkage Disequilibrium in a Spanish Common Bean Diversity Panel Revealed through Genotyping-by-Sequencing

**DOI:** 10.3390/genes9110518

**Published:** 2018-10-23

**Authors:** Ana Campa, Ester Murube, Juan José Ferreira

**Affiliations:** Plant Genetics, Area of Horticultural and Forest Crops, SERIDA, 33300 Asturias, Spain; emurube@serida.org (E.M.); jjferreira@serida.org (J.J.F.)

**Keywords:** *Phaseolus vulgaris*, common bean, diversity panel, GBS, association mapping

## Abstract

A common bean (*Phaseolus vulgaris*) diversity panel of 308 lines was established from local Spanish germplasm, as well as old and elite cultivars mainly used for snap consumption. Most of the landraces included derived from the Spanish common bean core collection, so this panel can be considered to be representative of the Spanish diversity for this species. The panel was characterized by 3099 single-nucleotide polymorphism markers obtained through genotyping-by-sequencing, which revealed a wide genetic diversity and a low level of redundant material within the panel. Structure, cluster, and principal component analyses revealed the presence of two main subpopulations corresponding to the two main gene pools identified in common bean, the Andean and Mesoamerican pools, although most lines (70%) were associated with the Andean gene pool. Lines showing recombination between the two gene pools were also observed, most of them showing useful for snap bean consumption, which suggests that both gene pools were probably used in the breeding of snap bean cultivars. The usefulness of this panel for genome-wide association studies was tested by conducting association mapping for determinacy. Significant marker–trait associations were found on chromosome Pv01, involving the gene *Phvul.001G189200*, which was identified as a candidate gene for determinacy in the common bean.

## Highlights

- A diversity panel (SDP) of 308 common bean lines was established for GWAS
- SDP gathered a wide genetic common bean diversity
- The main Spanish local diversity is represented in the SDP
- SDP is appropriate for the study of pod traits related to snap consumption

## 1. Introduction

The common bean (*Phaseolus vulgaris* L.) is one of the most important legumes for direct human consumption [1]. The common bean shows a broad phenotypic polymorphism, and both pods and seeds can be used for consumption depending on the genotype. Pods can be harvested before the seed development phase and consumed after cooking (green, French, or snap beans), while seeds can be harvested at physiological maturity (shell beans) or complete maturity (dry beans) and then consumed after re-hydrating and cooking. 

The common bean is a diploid species (2n = 2x = 22) native to America, where the wild forms are distributed from northern Mexico to northwestern Argentina [2]. The origin of the wild common bean remains a topic for debate. Recent works based on sequence data support a Mesoamerican origin of wild forms [3,4], from where the common bean expanded to South America resulting in two major ecogeographically and genetically distinct wild gene pools, the Andean (AN) and the Mesoamerican (MA) pools. Domestication took place after the formation of these gene pools in independent events [3,5,6,7,8,9,10,11,12]. The self-pollinating nature of the species and their geographical and ecological separation over millennia have combined to achieve marked differences between the two gene pools at the morphological [13,14,15,16] and molecular levels [3,5,10,17,18]. The common bean probably arrived in Europe through the Iberian Peninsula around the 16th century, after the exploration of the Americas [19]. Although the two main gene pools, AN and MA, were introduced into Europe, a prevalence of the AN type was detected based on seed proteins and molecular markers [20,21,22,23,24,25].

In the Iberian Peninsula, the common bean has been traditionally grown and consumed since its arrival. A wide phenotypic diversity has been described among a set of 296 local accessions collected in Spain in the middle of the 20th century [26]; different growth habits, pod phenotypes, types of use (snap, dry or both), and seed phenotypes ranging in size between 115 to 488 seeds in 100 g, have been described, although those having white seeds and being used as dry beans were the most common. Most of this Spanish diversity is preserved at the National Gene bank in the Center for Plant Genetic Resources (Centro de Recursos Fitogenéticos, CRF, Madrid), in which a common bean collection of more than 3500 accessions collected in Spain since 1978, is maintained. A core collection of 211 accessions (the Spanish Core Collection; SCC) was established from the CRF collection based on two criteria, geographical origin and seed phenotype [24,27,28]. The SCC was evaluated for morpho-agronomic traits and for resistance to different local pathogens, showing a wide genetic diversity for all traits [29,30,31]. The SCC was also evaluated with a small number of molecular markers, including variation in the major seed protein phaseolin [24], and results distinguished between the two main gene pools of the common bean, AN and MA. The SCC was later updated; it currently consists of 202 accessions. Massive genotyping of the updated SCC could supply data about the origin and relationships among these local materials, and about the diversity of the species and should contribute to its rationalization.

Advances in next-generation sequencing (NGS) technologies have provided fast and cost-efficient methods for DNA sequencing, to the point where genotyping-by-sequencing (GBS) technology is now feasible for species with high diversity and large genome [32]. Genotyping-by-sequencing generates a large number of single nucleotide polymorphism (SNP) datasets that can be physically positioned when a reference genome of the species is available (massive genotyping). In the common bean, the first chromosome-scale genome reference has been available since 2014 [33] and GBS is widely used for high-density linkage map construction, diversity analysis, and genome-wide association study (GWAS) [34,35,36,37,38] GWAS is a powerful tool for forward genetics analyses (from phenotype-to-genotype-to-genome) in which population samples consisting of individuals with a broad genetic variance (diversity panel) are screened for phenotypes of interest. At least two different diversity panels, consisting of mainly American genotypes, have been established in common bean: the AN Diversity Panel [35] and the MA Diversity Panel [39]. These diversity panels have been used to investigate the genetic architecture of important morpho-agronomic and nutritional traits.

One of the most important factors to be considered in a GWAS is the constitution of the diversity panel, including sample size, linkage disequilibrium (LD), population structure, or genetic relationship between individuals [40,41]. In the current work, a common bean diversity panel representing most of the Spanish diversity for this species was set up, including the updated SCC, and several international breeding lines, as well as old and elite cultivars. The objective of the work described here was to assess the structure and diversity of this panel for its use in GWAS and to investigate the origin and diversity of the local germplasm in order to maximize its conservation and use.

## 2. Material and Methods

### 2.1. Plant Material

**Figure 1:**
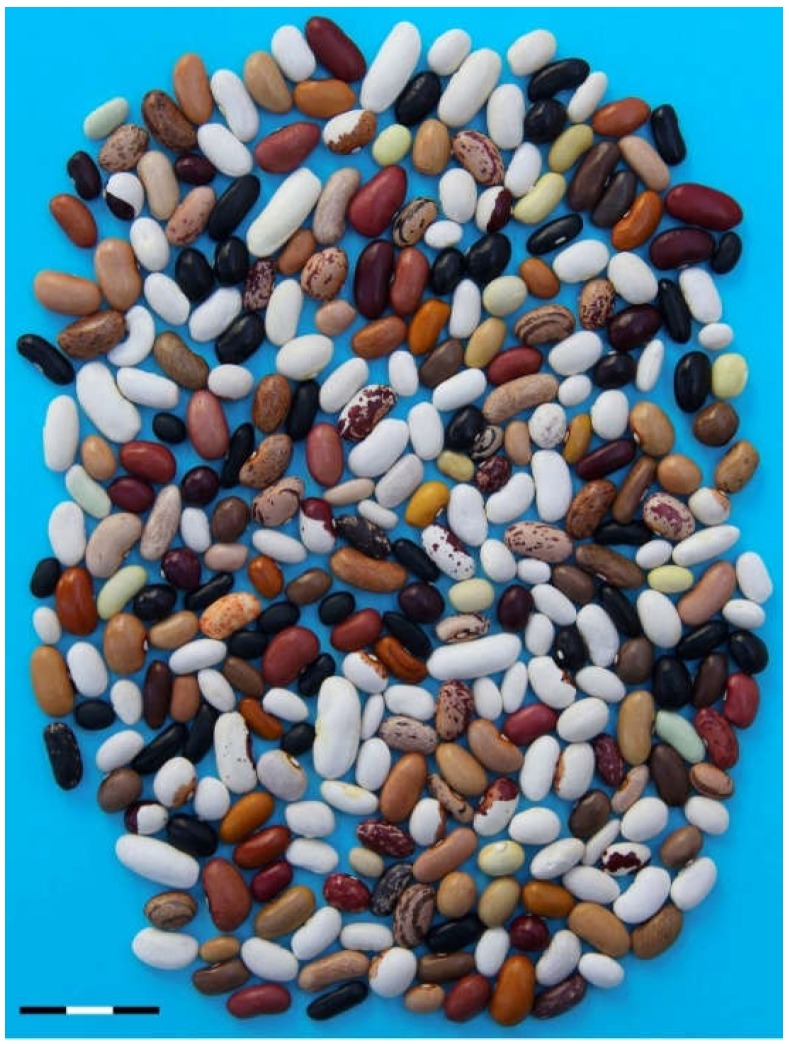
Phenotypic seed diversity included in the panel (one seed per line). Bar represents 3 cm.

A group of 308 *P. vulgaris* materials selected on the basis of type of material (landrace or elite cultivar), the form in which the bean is consumed (dry or snap), and previous genetic knowledge, was assembled into a panel, referred to as the SDP (Spanish Diversity Panel; Table S1). Lines (Figure 1) were obtained by selfing one plant per accession in a greenhouse located at the SERIDA (Regional Service for Agri-Food Research and Development) station in Asturias, northern Spain (43°29′01″ N, 5°26′11″ W; elevation 6.5 m). Plants were watered and fertilized for normal growth and maintained under natural light, environmental relative humidity, and moderate temperature (18–25 °C) during the year 2016. The SDP included 220 landraces, most of them from the updated SCC [24,27], and 51 elite cultivars, most of them cultivated in Europe for snap bean consumption, with the remaining 37 lines derived from traditional old cultivars and well-known breeding lines. The two sequenced bean genotypes, G19833, of AN origin [33], and BAT93, of MA origin [42], were included in the panel as references for the common bean gene pool.

### 2.2. DNA Isolation

Young leaves from one plant of each line were collected and DNA was isolated using the CTAB method [43] with modifications. Tissue was frozen in liquid nitrogen and pulverized. Concentrations of DNA were quantified photometrically (260–280 nm) using a Biomate 3 ultraviolet–visible spectrophotometer (Thermo Scientific, Waltham, MA, USA). The quality levels of the isolated DNA samples were verified in 1% agarose gels, stained with RedSafe (INtRON Biotechnology, Gyunggi-Do, Korea), and visualized under ultraviolet light. DNA samples were preserved at −80 °C.

### 2.3. Genotyping by Sequencing

Genotyping-by-sequencing, as described by Elshire et al. [32], was carried out at BGI-Tech (Copenhagen, Denmark) using the *ApeK*I restriction enzyme. A GBS sequencing library was prepared by ligating the digested DNA to unique nucleotide adapters (barcodes) followed by PCR with flow-cell attachment site tagged primers. Sequencing was performed using Illumina HiSeq4000 and 100x Paired-End. The sequencing reads from different genotypes were deconvoluted using the barcodes and aligned to the *Phaseolus vulgaris* L. v1 reference genome ([33]; Gene Bank Accession: GCF_000499845.1), using the Burrow Wheelers Alignment tool [44]. Single nucleotide polymorphism markers were extracted using the GBS pipeline implemented in TASSEL 5.2.39 software [45]. Data were filtered considering missing values (<5%), physical distance (>500 bp), and minor allele frequency (MAF > 0.01). The distribution of the SNPs along chromosomes was calculated with the qqman package [46] of the R project for statistical computing [47]. In order to estimate the rate of mistake in the GBS analysis two duplicated DNA samples of the landrace BGE025740 derived from the same isolation process were included. Rate of mistake was calculated the ratio of the differences between duplicated samples by the total number of SNPs.

### 2.4. Linkage Disequilibrium

Linkage disequilibrium was estimated by calculating the square value of correlation coefficient (*r^2^*) between pairs of markers [48] using the TASSEL 5.2.39 software [45]. A threshold of *r*^2^ ≥ 0.5 was considered to indicate LD. The level of LD was estimated for the entire panel and for the specific subgroups identified with Structure v2.3.4. Within these subgroups, LD was calculated considering only the polymorphic set of markers in each case. *p*-values for each *r*^2^ estimate were obtained with a two-tailed Fisher’s exact probability test and a threshold of *p* < 0.0001 was considered significant. Linkage disequilibrium patterns per chromosome were also calculated.

### 2.5. Data Analysis

Population structure was evaluated using Structure v2.3.4 [49] and Structure Plot v2 software [50]. The STRUCTURE parameters used were an admixture model with independent allele frequencies, a burn-in period of 1000 and 5000 Markov Chain Monte Carlo (MCMC) iterations, with 20 replications for each hypothetical number of subpopulations (*K*) between 1 and 4. The optimum *K* value value was calculated according to Evanno et al. [51]. A new burn-in period of 10,000 and 30,000 MCMC iterations was conducted for the optimum *K* value to assign accessions to subpopulations.

Cluster analysis was conducted with the FactoMineR [52] package of the R project, considering the Euclidean distance and the Unweighted Pair Group Method with Arithmetic Mean (UPGMA) agglomeration method. The FactoMineR package was also used to compute a Principal Component Analysis (PCA). The contribution of each SNP in the final PCA plot was visualized with the function fviz_contrib () of factoextra [53] package.

Functional annotations of specific chromosome regions were studied using Ensembl Plants resource [54]). To understand biological meaning behind a list of genes the Database for Annotation, Visualization, and Integrated Discovery (DAVID) v6.8 was used [55,56]).

### 2.6. Genome-Wide Association Study for Determinacy

Association mapping for determinacy was conducted to evaluate the utility of the SDP for GWAS. Phenotyping was performed at the same time as the lines were grown in the greenhouse. Growth habit was characterized as being either determinate (main stem ending in a terminal flower bud) or indeterminate (the flower bud was not terminal). Association studies were conducted using the generalized lineal model (GLM) and the mixed linear model (MLM) implemented in TASSEL 5.2.39 software [45]. The GLM is appropriate for variables that are not normally distributed [41], and it is based on *P* + *Q* matrices, where *P* is the phenotype matrix and *Q* is the population structure matrix from PCA. The MLM includes both fixed and random effects and it is based on the equation

*Y* = *X*α + *P*β + *K*μ + e

where *Y* is phenotype, *X* is genotype, *P* is the PCA matrix, both *X* and *P* represent fixed effects, *K* is the relative kinship matrix value, and e is for residual effects. The Bonferroni correction for α 0.001 (−Log(*p*) = 6.5) was used for the identification of significant SNP markers. Manhattan plots and QQ plots were developed using the qqman package in R [46,47].

## 3. Results

### 3.1. Genotyping

**Figure 2:**
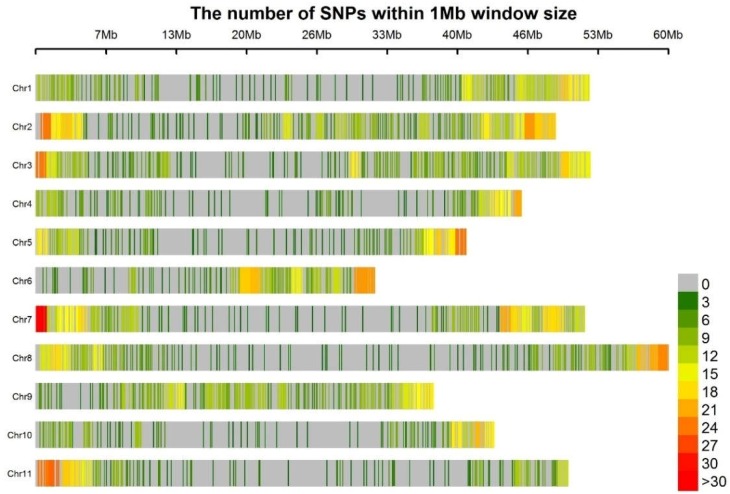
Distribution along the eleven bean chromosomes of the 3099 single nucleotide polymorphisms (SNPs).

Sequencing of the GBS libraries yielded approximately 2.58 million reads per line, the Q20 value of each sample was above 98% and most of the sample mapping rate was >83%. A total of 9070 mapped SNPs were identified. The error rate of this analysis was estimated to be 0.13% by comparing the duplicated DNA samples from BGE025740. After filtering for missing values, physical distance, and minor allele frequency, a total of 3099 SNPs distributed among the eleven common bean chromosomes was selected (Figure 2; Table S2). The average number of SNPs per chromosome was 282, ranging from 179 on chromosome Pv10 to 399 on chromosome Pv02. For all chromosomes, fewer SNPs were identified in the regions around centromeres than in the regions around telomeres. The average distance between SNPs was 0.17 mega base pair (Mbp), with a minimum distance of 501 bp on chromosome Pv04 and a maximum distance of 3.99 Mb on chromosome Pv10.

### 3.2. Population Structure

**Figure 3:**
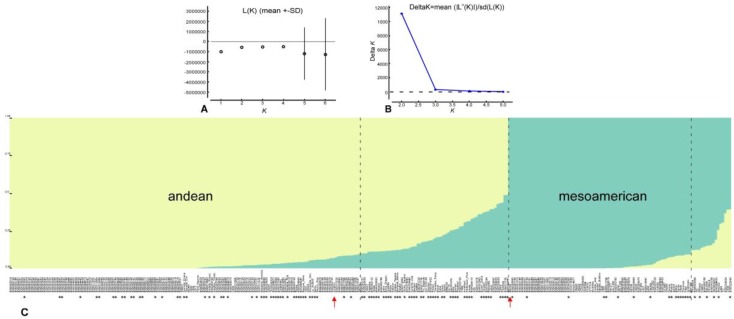
(**A**) Mean *L*(*K*) (±SD) over 20 runs for each *K* value. (**B**) Plot of ∆*K*. (**C**) Plot of ancestry estimated for *K* = 2. Bars represent the estimated membership coefficients for each accession in each population (represented by different colors) using a threshold value of 0.9 for the Q statistic. Arrows indicate the Andean cultivar G19833 and the Mesoamerican BAT93. Asterisks indicate lines that can be used for snap consumption.

A hypothetical number of subpopulations between two and four were tested with Structure v2.3.4 (Figure S1). The ∆*K* value indicated an optimal number of subpopulations of two (Figure 3A,B). At *K* = 2 and a threshold of 0.9 for Q statistics, two main groups are identified (Figure 3C): a group of 216 lines closely related to G19833, of AN origin, and a group of 92 lines closely related to BAT93, of MA origin. However, a total of 82 lines showed recombination between the two main gene pools. Lines with potential use as snap beans were observed in both groups, but principally they showed recombination. Only three lines derived from the elite snap bean cultivars did not show recombination between the two gene pools: ‘Garrafal Oro’ and ‘Garrafal Enana’ assigned to the AN group, and ‘Helda’ assigned to MA. With regard to lines derived from the SCC, they were assigned to the two main groups, MA and AN, and 30 of them (approximately 14%) showed recombination.

### 3.3. Linkage Disequilibrium

Linkage disequilibrium level was calculated for the entire panel and separately within the subgroups of 148 AN lines, 78 MA lines, and 82 recombinant lines (Table 1). Only 10.11% of the total panel showed significant LD. LD levels were very low, less than 1% in the AN, MA, and recombinant subgroups. For this reason, LD patterns were only calculated for the total panel (Table 2). Chromosomes Pv01, Pv03, Pv09, and Pv11 showed the highest percent of intrachromosomal LD (>20%), coinciding with regions around centromeres (Figure S2). Chromosome Pv09 showed the largest interchromosomal LD, with more than 20% LD with chromosomes Pv01, Pv03, Pv07 and Pv11.

### 3.4. Unweighted Pair Group Method with Arithmetic Mean Clustering

**Figure 4:**
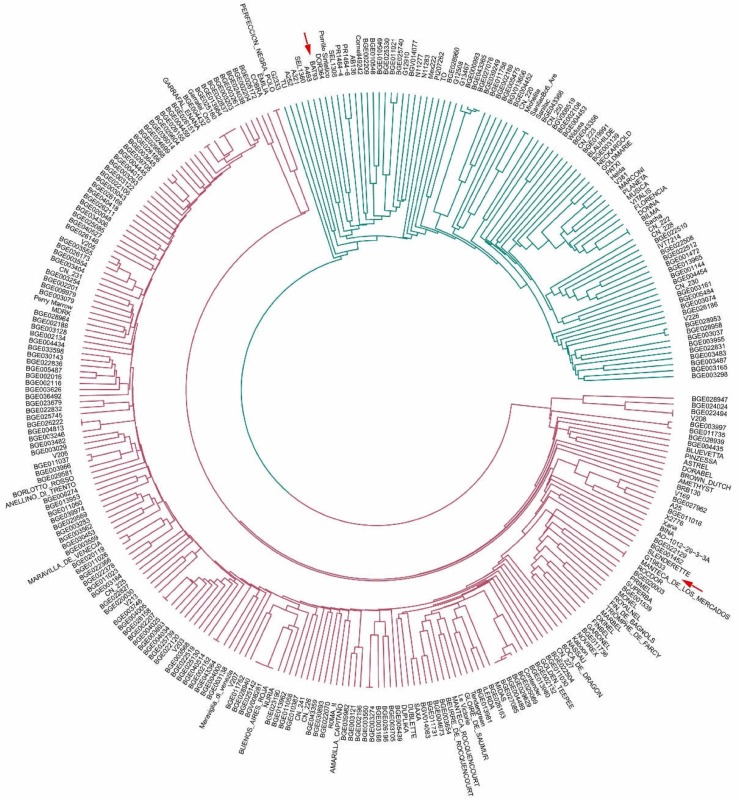
Circular phylogenetic tree obtained for 308 lines and 3099 SNPs. The two colors identify the two main groups observed. Arrows indicate the Andean cultivar G19833 and the Mesoamerican BAT93.

To characterize the relatedness among the 308 bean lines, a dendrogram was constructed using the UPGMA method and 3099 SNPs. Figure 4 shows the circular phylogenetic tree obtained. Two main groups were observed, one including the MA line BAT93 and the other the AN line G19833. Most lines (70%) were clustered within the AN gene pool.

Lines showing the same genetic profile for the 3099 SNPs were observed in both groups. The MA cluster had three groups of materials showing the same profile: lines derived from accessions BGE027076-BGE039249, lines derived from Sanilac-SanilacBC6_Are, and lines derived from elite cultivar Bilma-Sacha. Within the AN gene pool three groups of materials also showed the same profile:

(i) Lines derived from commercial elite cultivars for snap bean consumption: ‘Perfeccion Negra Polo’ and ‘Emilia’, ‘Manteca de los Mercados’, and ‘Rocdor’;
(ii) lines derived from accessions included in the SCC: BG026222-BGE004813, BGE026158-BGE002207, BGE040514-BGE002152, BGE003550-BGE003274, and BGE029629-BGE004489-BGE027085, BGE022494-V208;
(iii) lines derived from commercial elite cultivars for snap bean consumption were included in the SCC, ‘Garrafal enana’-BGE026151, ‘Garrafal Oro’-BGE025180-BGE013964-BGE022837, and ‘Buenos Aires Roja’-BGE028940-BGE025142-BGV008281.

### 3.5. Principal Component Analysis

**Figure 5:**
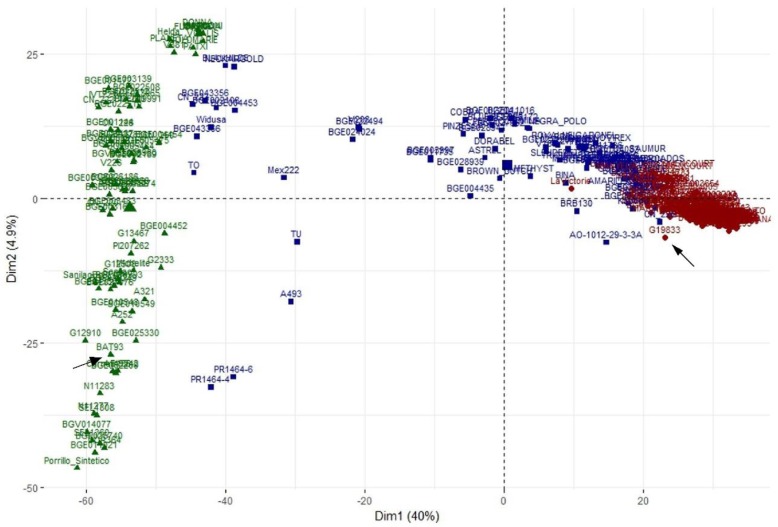
Two-dimension plot obtained from Principal Component Analysis (PCA) for 308 lines and data of 3099 SNPs. Lines are colored according to the structure analysis for *K* = 2: green indicates lines included in the Mesoamerican group, red indicates lines included in the Andean group, and blue indicates lines showing admixture between both groups. Arrows indicate the location of the Andean cultivar G19833 and the Mesoamerican BAT93.

Figure 5 shows the two-dimensional plot obtained in the PCA analysis. The first component (Dim1) accounted for 40% of the variance and distinguished between the two main groups, AN and MA. The recombinant lines identified with STRUCTURE clustered at the intersection between these two main groups. The second principal component (Dim2) accounted for only 4.9% of the variance but revealed more diversity within the MA group than within the AN, in which many accessions occupied a similar position in the plot.

Among the 3099 SNPs, the ones showing the greatest contribution to the Dim1 and Dim2 of the PCA were selected. The PCA obtained with the 15 more influent SNPs showed the separation between the MA and the AN groups, as well as most of the recombinant lines (Figure S3). These 15 SNPs involved five chromosomes: Pv01, Pv04, Pv07, Pv08, and Pv09 (Table S3). Most of the 15 SNPs were located in coding regions, one was located in a 3′-untranslated region, three were in introns, and only one was located in an intergenic region. Even though most of the 15 most influential SNPs were located in coding regions, the difficulty in assigning a SNP to a causative gene is well documented [57]. A candidate gene search, centered on the 100-kb region surrounding each significant SNP, was carried out. Using this approach, some chromosome regions overlapped, so that a total of eight regions involving chromosomes Pv01, Pv04, Pv07, Pv08, and Pv09 were considered (Table S4). Using the Ensembl Plants tool, a total of 318 genes were annotated in these regions (Table S5). According to the candidate genes associated with domestication proposed by Schmutz et al. [33], these regions included 60 of the 1835 MA genes, eight of the 748 AN genes, and three genes associated with both gene pools (Table S5).

### 3.6. Utility of the Spanish Diversity Panel for Genome-Wide Association Study

Association analysis for determinacy was performed on the SDP in combination with the 3099 SNPs (Figure S4). GLM analysis revealed a total of 16 SNPs significantly associated with determinacy on chromosome Pv01, between the physical positions 6–45 Mbp (Table S6). In the MLM analysis only one SNP located at 37 Mbp on chromosome Pv01 was significantly associated with determinacy (Table S6).

## 4. Discussion

### 4.1. Genetic Diversity and Origin

In this work, a common bean diversity panel (SDP) of 308 lines, that included accessions representing the main local Spanish diversity, elite cultivars, and breeding lines, was established. The SDP was genotyped through GBS which supplied a total of 9070 SNPs; even though only 3099 SNPs distributed along the eleven bean chromosomes were used in the analysis.

This panel contained 202 lines derived from the SCC, so the results obtained in this work constitute the deepest molecular characterization conducted to date on this core collection and will contribute to maximizing its conservation and use. The accessions maintained in the SCC can be considered landraces as they were gathered in different collecting missions performed around Spain since 1970, including in areas where small farmers selected and maintained their own cultivars [24,27]. Spanish Diversity Panel also contains snap bean elite cultivars obtained from breeding programs, which offers the opportunity to investigate their relationships with materials classified as landraces in gene banks. For example, no differences in SNP profile were detected between the old cultivar ‘Garrafal Oro’ and the accessions BGE025180, BGE013964, and BGE022837 or between the old cultivar ‘Buenos Aires Roja’ and the accessions BGE028940, BGE025142, and BGV008281. This finding suggests that some accessions which were considered to be landraces probably derived from commercial/elite cultivars after several years of maintenance by farmers, and reflects the difficulty in differentiating between the two types of materials. In fact, both cultivars ‘Garrafal Oro’ and ‘Buenos Aires Roja’ are old cultivars that have already been described in the Spanish fields in 1960 [26]. This result is to be expected, because the presence of elite cultivars, mainly for snap bean consumption, derived from bean breeding programs in Europe which began at the end of the 19th century [58]. Moreover, *P. vulgaris* is a highly self-pollinated species so local farmers frequently use their own seed for planting, with the maintenance of cultivars being quite straightforward. This work also reflects the important genetic diversity present within elite cultivars, because they are an important source of genes for a species.

### 4.2. Population Structure

Structure, cluster, and PCA analysis based on the 3099 SNPs showed the existence of two main groups of germplasm corresponding to the AN and MA gene pools, although most lines (70%) were attributable to the AN gene pool. This result agrees with previous works in which the AN gene pool was prevalent within the European material [20,21,22,23,25] and also within the Spanish germplasm [24]. However, the MA group of materials analyzed in this work showed a greater genetic diversity than the AN group, as had been reported in previous studies [12,33,35,59,60]. A higher level of diversity in the MA gene pool than in the AN gene pool was also found in the wild forms [10,61]. Based on sequence data, Bitocchi et al. [4] proposed that, before the domestication of the common bean, there was a severe genetic bottleneck in the AN wild populations, which could explain the narrower AN diversity.

Even though two main subpopulations were identified in the structure analysis, 82 lines showed introgression between the two gene pools. This finding agreed with the high proportion (approximately 44%) of European common bean germplasm that is estimated to be derived from hybridization between the two gene pools (Angioi et al. 2010). In the present work, approximately 14% of the Spanish landraces showed recombination between the two gene pools, a finding which is in agreement with the results of Angioi et al. [25], who observed an uneven distribution of hybrids around Europe, with low frequencies in Spain and Italy. Interestingly, most of those recombinant lines are cultivated for snap bean consumption (see Table S1), which suggests that it is likely both gene pools were used in the breeding of snap bean cultivars. Lines for snap consumption which showed no recombination between the two gene pools were also detected. Of particular interest are the old snap bean cultivars ‘Garrafal Oro’ and ‘Garrafal Enana’ assigned to the AN group, and ‘Helda’ to the MA one. The origin of cultivars for snap bean consumption is not clear. They are thought to have been mainly derived from dry beans after introgression in Europe, where they were rapidly consolidated as a new crop [62]. Accordingly to Brown et al. [63] and Gepts et al. [5] they are predominantly of AN origin but Blair et al. [64] suggests a MA origin. It is important to note that genetic diversity is a concept that depends on the type and number of molecular markers used and it is influenced by sampling effects, so comparison between results from different studies can be difficult.

The identification of duplicated materials in germplasm resources was not cost-effective until the development of the sequencing technologies. In this work, redundant material has been identified based on the 3099 SNPs. This information can be useful for the optimization of the SCC, as redundancy is one of the main problems facing germplasm collections, which consumes gene bank resources. On the other hand, an optimized SDP without the presence of redundant genotypes should be considered for future GWAS.

### 4.3. Linkage Disequilibrium

Linkage disequilibrium is the nonrandom association of alleles at distinct loci in the genome of a sampled population [48] and constitutes the basis for association mapping approaches. Linkage disequilibrium is highly population-specific and can determine the utility of a panel for GWAS [41]. Based on the 3099 SNPs used in this work, only 10% of the pairwise LD comparisons were in disequilibrium, even when a very restrictive threshold of *r*^2^ ≥ 0.5 was taken into account. Concerning the distribution of LD patterns, chromosomes Pv01, Pv03, Pv09, and Pv11 showed the highest percentage of intrachromosomal LD (>20%), coinciding with highly conserved centromeric regions. This is to be expected as LD has been shown to be noticeably elevated (~5 Mb) in centromeres and other heterochromatic regions, as well as in duplicated regions of the genome [65]. For interchromosomal LD, chromosome Pv09 showed the highest percentage of LD (>20% LD) with chromosomes Pv01, Pv03, Pv07, and Pv11. Different LD patterns could also be related to the independent domestication events for the MA and AN gene pools, in which different chromosomes regions were indirectly selected. According to Schmutz et al. [33], chromosomes Pv02, Pv07, and, in particular, chromosome Pv09 showed signatures of selection in the MA population, whereas the Andean domestication event primarily involved chromosomes Pv01, Pv02, and Pv10. Results of the PCA analysis support the proposal that chromosome Pv09 plays an important role in the differentiation of the two main common bean gene pools. Among the 15 SNP markers that showed the greatest contribution to the differentiation between the MA and the AN groups of the SDP, five are located on chromosome Pv09, involving regions in which 50 candidate genes associated with domestication have been described ([33], see Table S3).

### 4.4. Genome-Wide Association Study

To evaluate the utility of the SDP, an association mapping analysis for the well-known morphological trait determinacy was conducted. The *fin* gene is involved in the genetic control of this trait, with recessive genotypes controlling the determinate growth habit. This locus was mapped to the end of chromosome Pv01 and a candidate gene (*PvTFL1y*, *PHAVU_001G189200g*; Pv01:45,561,512..45,563,326) has been reported from homology with the *TFL1y* gene of *Arabidopsis thaliana* L. [66,67]. In the GLM-GWAS a significant determinacy-associated region was identified on chromosome Pv01, from 6 to 45 Mbp. The candidate gene for determinacy, *Phvul.001G189200*, was identified in the GLM-GWAS, although the chromosome region identified spread to 6 Mbp of chromosome Pv01. This could be explained by the strong LD block identified in this region of chromosome Pv01, which evince the importance of considering the distribution of LD in each panel for the interpretation of GWAS results. Concerning MLM-GWAS, a significant determinacy-associated SNP was only identified on chromosome Pv01, in the position 37 Mbp. The region of 45 Mbp, in which the candidate gene, *PHAVU_001G189200g*, has been located, was not identified in the MLM-GWAS. Determinacy is a complex trait that has been selected during domestication of common bean, and leguminous crops in general [68]. Multiple origins have been proposed for determinacy in common bean based on the broad mutational spectrum observed in *PvTFL1y*, including retrotransposon insertion and deletion [69], so the possibility of other genes apart from *PvTFL1y* involved in the genetic control of determinacy cannot be discarded.

## 5. Conclusions

In this work, a diversity panel of 308 common bean lines (SDP) was established and genotyped with 3099 SNPs obtained through GBS. Broad genetic and morphological diversity was observed in the SDP. Most of the landraces included were derived from the Spanish common bean core collection, so this panel can be considered to be representative of the local Spanish diversity for this species. SDP also contains snap bean elite cultivars obtained from breeding programs, so it is appropriate for the study of pod morphological traits related to snap bean consumption. Some groups of accessions with the same profile from the 3099 SNPs were identified, suggesting the possibility of removing some duplicate accessions in order to maximize panel diversity. Information concerning redundant accessions can be useful for the management of the Spanish local diversity maintained in gene banks. Close relationships between lines derived from landraces and old cultivars were identified, revealing the difficulty of differentiating between both types of materials. The usefulness of SDP for future GWAS was validated though the association mapping of determinacy.

## Figures and Tables

**Table 1:** Pairwise linkage disequilibrium (LD) analysis for polymorphic loci calculated in the whole Panel, and in the subgroups Andean, Mesoamerican, and recombinants. A threshold of *r*^2^ ≥ 0.5 was considered as LD.

Group	n	No Polymorphic SNPs	Pairwase LD		
			Average *r*^2^	%*LD*	%Significant *LD*
Total Panel	308	3099	0.18	10.11	100
Andean	148	2152	0.03	0.95	92.61
Mesoamerican	78	2596	0.05	0.92	90.2
Recombinants	82	2994	0.06	1.08	89.2

**Table 2:** Linkage disequilibrium pattern in the Spanish Diversity Panel (SDP). Average value for *r*^2^ is indicated. %LD indicates the percentage of SNP pairs in linkage disequilibrium (*r*^2^ ≥ 0.5).

		Chr Pv01	Chr Pv02	Chr Pv03	Chr Pv04	Chr Pv05	Chr Pv06	Chr Pv07	Chr Pv08	Chr Pv09	Chr Pv10	Chr Pv11
Chr Pv01	*r* ^2^	0.3										
	%LD	29.4%										
Chr Pv02	*r* ^2^	0.15	0.18									
	%LD	4%	9.30%									
Chr Pv03	*r* ^2^	0.23	0.15	0.24								
	%LD	18.7%	3.3%	20.3%								
Chr Pv04	*r* ^2^	0.15	0.13	0.15	0.16							
	%LD	6.4%	3.3%	5.7%	10%							
Chr Pv05	*r* ^2^	0.19	0.13	0.17	0.13	0.19						
	%LD	12.3%	2.7%	8.2	3.1%	11.7%						
Chr Pv06	*r* ^2^	0.21	0.14	0.19	0.13	0.17	0.24					
	%LD	13.3%	2%	10.9%	3.4%	6.2%	16.4%					
Chr Pv07	*r* ^2^	0.2	0.14	0.19	0.14	0.15	0.18	0.21				
	%LD	15.5%	3.1%	13.4%	4.5%	6.7%	10.3	16%				
Chr Pv08	*r* ^2^	0.18	0.14	0.18	0.14	0.15	0.17	0.17	0.19			
	%LD	11.7%	4.10%	10.1%	6.4%	6.5%	7.50%	8.7%	13.1%			
Chr Pv09	*r* ^2^	0.26	0.18	0.24	0.17	0.19	0.22	0.24	0.21	0.35		
	%LD	24.7%	6.4%	21.6%	6.7%	10.4%	16.4%	21.2%	14%	35.4%		
Chr Pv10	*r* ^2^	0.13	0.10	0.14	0.09	0.11	0.13	0.13	0.11	0.17	0.14	
	%LD	5.5%	0.9%	5.9%	0.01%	0.02%	3.5%	4.8%	2.2%	8.4%	7%	
Chr Pv11	*r* ^2^	0.22	0.15	0.20	0.15	0.17	0.18	0.19	0.18	0.24	0.13	0.25
	%LD	19.4%	3.8%	15.1%	6.2%	9.5%	10.6%	13.7%	11.7%	22.1%	4.2%	21.4%

## Supplementary Materials

The following are available online at http://www.mdpi.com/2073-4425/9/11/518/s1. Table S1: List of accessions included in the Spanish Diversity Panel, Table S2: Distribution of the 3099 SNPs, Table S3: Tag sequences, Table S4: Regions of 100-kb surrounding each one of the most influent SNPs, Table S5: Genes annotated in the Regions of 100-kb surrounding each one of the most influent SNPs, Table S6: Determinacy-associated SNPs, Figure S1: Plot of ancestry, Figure S2: Linkage disequilibrium plots, Figure S3: PCA Plot, Figure S4: Manhattan plots.
