# Supplementary material for: Genetic Diversity, Population Structure, and Linkage Disequilibrium in a Spanish Common Bean Diversity Panel Revealed through Genotyping-by-Sequencing
Source: Genes (Basel). 2018 Oct 23;9(11):518. doi: 10.3390/genes9110518 (PMC6266623; doi:10.3390/genes9110518)
Supplement: Supplementary file 1 [file genes-09-00518-s001.zip › Table_S4_R2.docx]

**Table S4**. **Regions of 100-kb surrounding each one of the most influent SNPs**. The 8 regions considered for the candidate gene search are indicated

| SNP | Chr | position (bp) | 100 kb upstream | 100 kb downstream | Region (Chr: start..end) |
| --- | --- | --- | --- | --- | --- |
| s1_6033703 | Pv01 | 6033703 | 5933703 | 6133703 | region I (Pv01: 5933703..6297061) |
| s1_6197061 | Pv01 | 6197061 | 6097061 | 6297061 |  |
| s4_43950011 | Pv04 | 43950011 | 43850011 | 44050011 | region II (Pv04: 43850011..44050011) |
| s7_4755652 | Pv07 | 4755652 | 4655652 | 4855652 | region III (Pv07: 4655652..4855652) |
| s7_5756712 | Pv07 | 5756712 | 5656712 | 5856712 | region IV (Pv07: 5656712..5987308) |
| s7_5831598 | Pv07 | 5831598 | 5731598 | 5931598 |  |
| s7_5887308 | Pv07 | 5887308 | 5787308 | 5987308 |  |
| s8_4345326 | Pv08 | 4345326 | 4245326 | 4445326 | region V (Pv08: 4245326..4966955) |
| s8_4499339 | Pv08 | 4499339 | 4399339 | 4599339 |  |
| s8_4866955 | Pv08 | 4866955 | 4766955 | 4966955 |  |
| s9_11995387 | Pv09 | 11995387 | 11895387 | 12095387 | region VI (Pv09: 11895387..12907237) |
| s9_12807237 | Pv09 | 12807237 | 12707237 | 12907237 |  |
| s9_13780397 | Pv09 | 13780397 | 13680397 | 13878576 | region VII (Pv09: 13680397..14078576) |
| s9_13978576 | Pv09 | 13978576 | 13880397 | 14078576 |  |
| s9_20452212 | Pv09 | 20452212 | 20352212 | 20552212 | region VIII (Pv09: 20352212..20552212) |
