# Supplementary material for: Genetic Diversity, Population Structure, and Linkage Disequilibrium in a Spanish Common Bean Diversity Panel Revealed through Genotyping-by-Sequencing
Source: Genes (Basel). 2018 Oct 23;9(11):518. doi: 10.3390/genes9110518 (PMC6266623; doi:10.3390/genes9110518)
Supplement: Supplementary file 1 [file genes-09-00518-s001.zip › Table_S6_R2.docx]

**Table S6.** **Determinacy-associated SNPs.** SNPs significantly associated with determinacy in the GWAS using both GLM and MLM. *R^2^* is the proportion of variability explained by the significant SNP for a particular marker-trait association test

| SNP name | Chr | Position (bp) | -Log(p) | *R^2^(%)* |
| --- | --- | --- | --- | --- |
| GLM |  |  |  |  |
| s1_6954979 | Pv01 | 6954979 | 6,56 | 4.0 |
| s1_23794365 | Pv01 | 23794365 | 10,77 | 6.9 |
| s1_28222218 | Pv01 | 28222218 | 8,19 | 5.4 |
| s1_36653147 | Pv01 | 36653147 | 10,92 | 7.0 |
| s1_36801972 | Pv01 | 36801972 | 6,99 | 4.4 |
| s1_37888758 | Pv01 | 37888758 | 16,72 | 10.5 |
| s1_40711009 | Pv01 | 40711009 | 6,64 | 4.1 |
| s1_44119818 | Pv01 | 44119818 | 6,54 | 4.1 |
| s1_44120921 | Pv01 | 44120921 | 7,29 | 4.6 |
| s1_44508726 | Pv01 | 44508726 | 8,28 | 5.5 |
| s1_44550650 | Pv01 | 44550650 | 7,13 | 4.4 |
| s1_44587700 | Pv01 | 44587700 | 7,97 | 5.2 |
| s1_44589925 | Pv01 | 44589925 | 7,46 | 4.9 |
| s1_45296322 | Pv01 | 45296322 | 7,76 | 5.0 |
| s1_45447965 | Pv01 | 45447965 | 7,24 | 4.6 |
| s1_45461809 | Pv01 | 45461809 | 8,37 | 5.4 |
| MLM |  |  |  |  |
| s1_37888758 | Pv01 | 37888758 | 8.07 | 12.4 |
