# Supplementary material for: Genetic Diversity, Population Structure, and Linkage Disequilibrium in a Spanish Common Bean Diversity Panel Revealed through Genotyping-by-Sequencing
Source: Genes (Basel). 2018 Oct 23;9(11):518. doi: 10.3390/genes9110518 (PMC6266623; doi:10.3390/genes9110518)

**Fig S1.** **Plot of ancestry.** Plot of ancestry estimated for *K*=2, *K*=3 and *K*=4. Bars represent the estimated membership coefficients for each accession in each population (represented by different colors) using a threshold value of 0.9 for Q statistic.


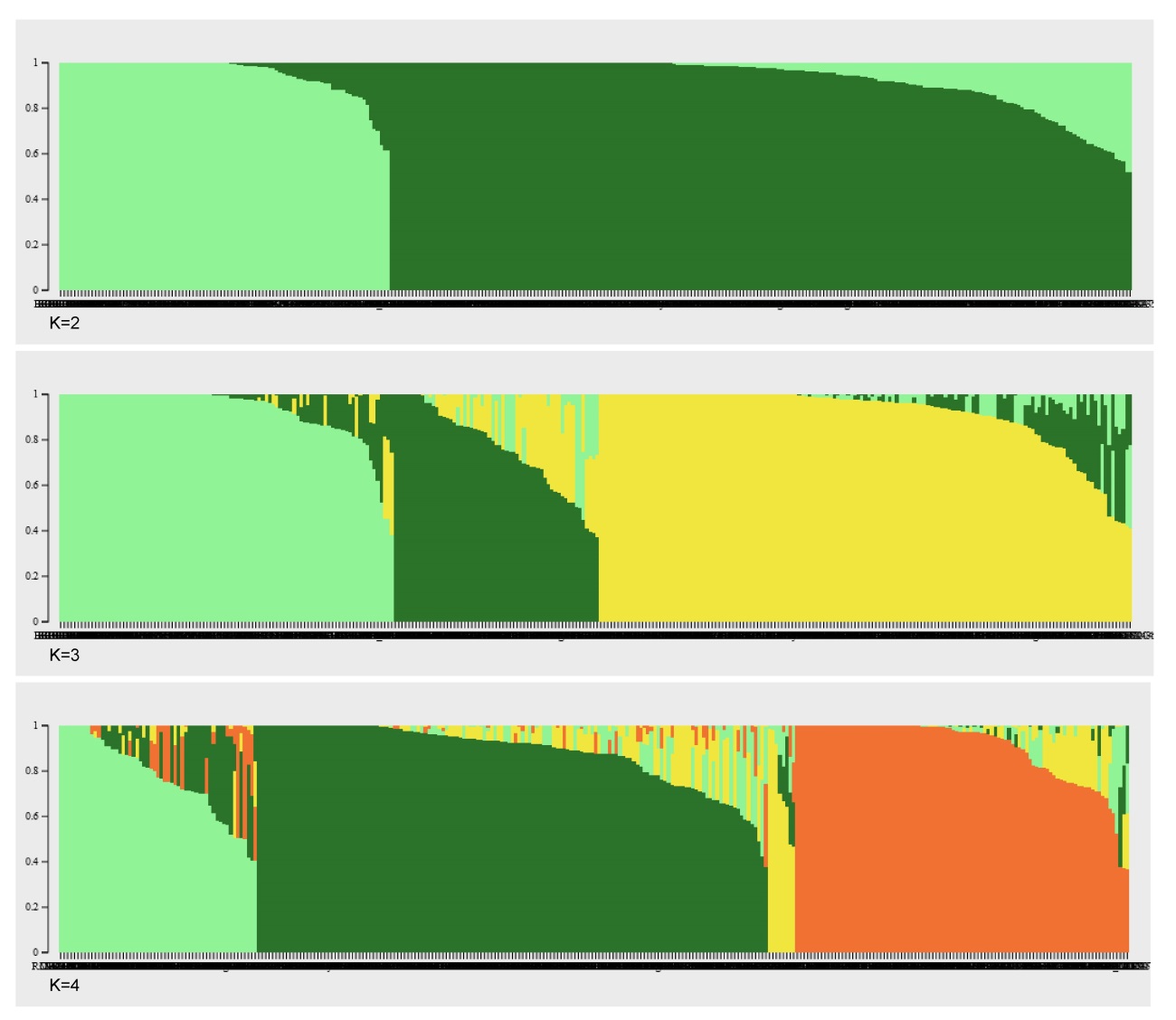

Supplement: Supplementary file 1 [file genes-09-00518-s001.zip › Fig_S1_R2.docx]
