# Supplementary material for: Genetic Diversity, Population Structure, and Linkage Disequilibrium in a Spanish Common Bean Diversity Panel Revealed through Genotyping-by-Sequencing
Source: Genes (Basel). 2018 Oct 23;9(11):518. doi: 10.3390/genes9110518 (PMC6266623; doi:10.3390/genes9110518)

**Fig.S2. Linkage disequilibrium plots**. Linkage disequilibrium (LD) plots for chromosomes Pv01, Pv03, Pv09 and Pv11 obtained from the LD analyses conducted in the total Panel. The black diagonal represents LD between each site and itself. Black bars in the diagonal represent centromeric regions. *r^2^* values are in the upper right and *p*-values in the lower left.


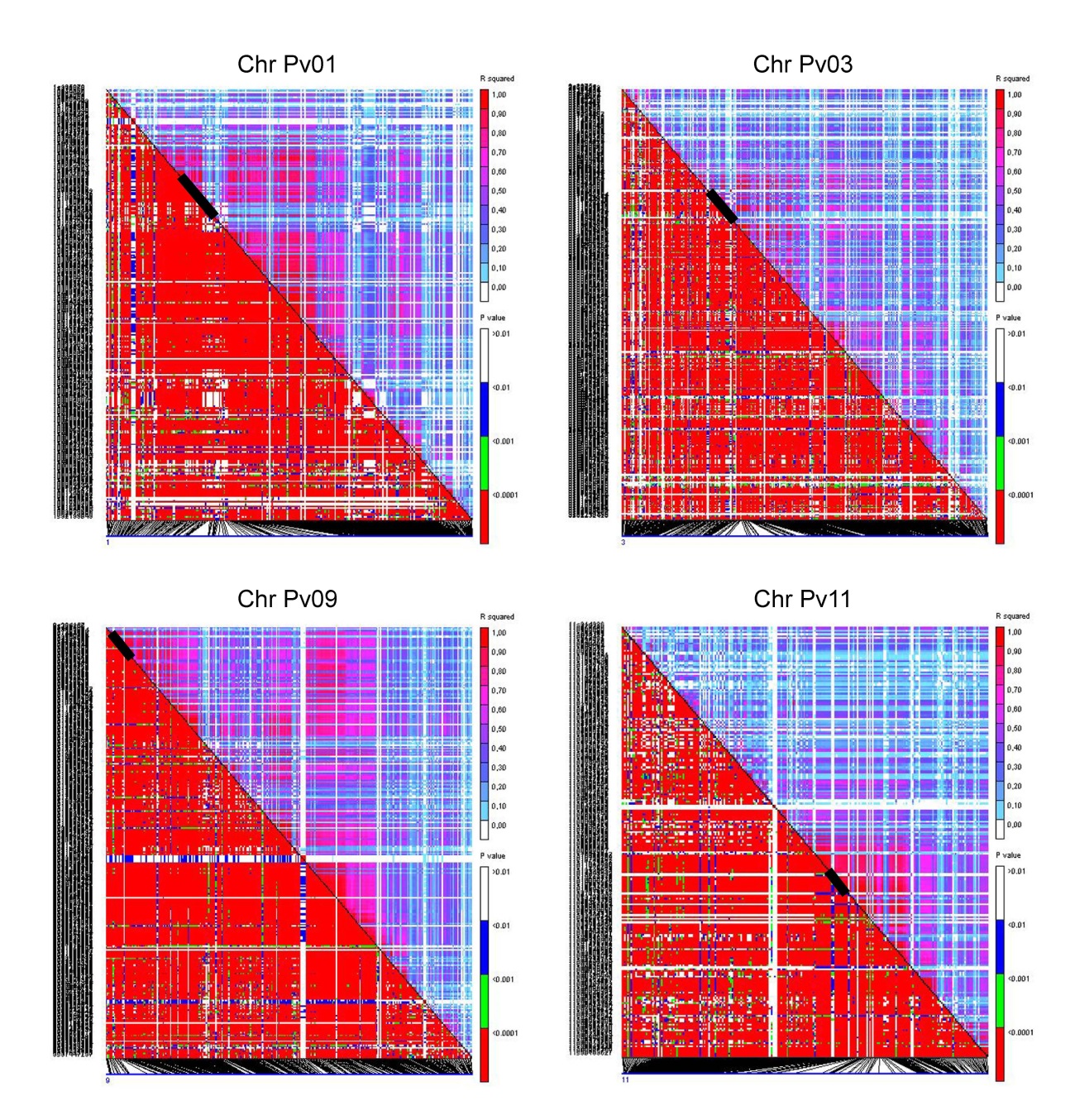

Supplement: Supplementary file 1 [file genes-09-00518-s001.zip › Fig_S2_R2.docx]
