# Supplementary material for: Genetic Diversity, Population Structure, and Linkage Disequilibrium in a Spanish Common Bean Diversity Panel Revealed through Genotyping-by-Sequencing
Source: Genes (Basel). 2018 Oct 23;9(11):518. doi: 10.3390/genes9110518 (PMC6266623; doi:10.3390/genes9110518)
Supplement: Supplementary file 1 [file genes-09-00518-s001.zip › Fig_S3_R2.docx]

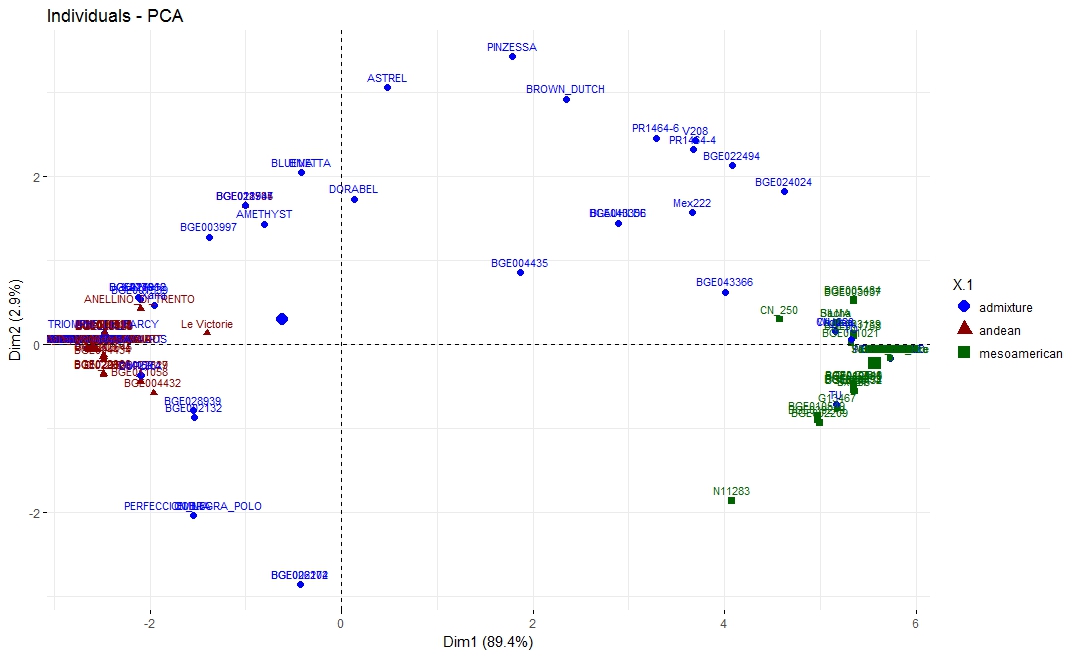


**Fig. S3**. **PCA Plot.** Two-dimension plot obtained from PCA for 308 lines and data of 15 SNPs. Lines are colored according to the Structure analysis for K=2: green indicates lines included in the Mesoamerican group, red indicates lines included in the Andean group; and blue indicates lines showing admixture between both groups.
