# Supplementary material for: Genetic Diversity, Population Structure, and Linkage Disequilibrium in a Spanish Common Bean Diversity Panel Revealed through Genotyping-by-Sequencing
Source: Genes (Basel). 2018 Oct 23;9(11):518. doi: 10.3390/genes9110518 (PMC6266623; doi:10.3390/genes9110518)
Supplement: Supplementary file 1 [file genes-09-00518-s001.zip › Fig_S4_R2.docx]

**Figure S4. Manhattan plots.** GWAS for determinacy run in the SDP genotyped with 3099 SNP. A) Manhattan and Q-Q plot plot obtained using GLM-GWAS B) Manhattan and Q-Q plot obtained using MLM-GWAS. Horizontal line is the Bonferroni cut-off for significance. Red points indicate the SNPs significantly associated to the morphological trait.

**
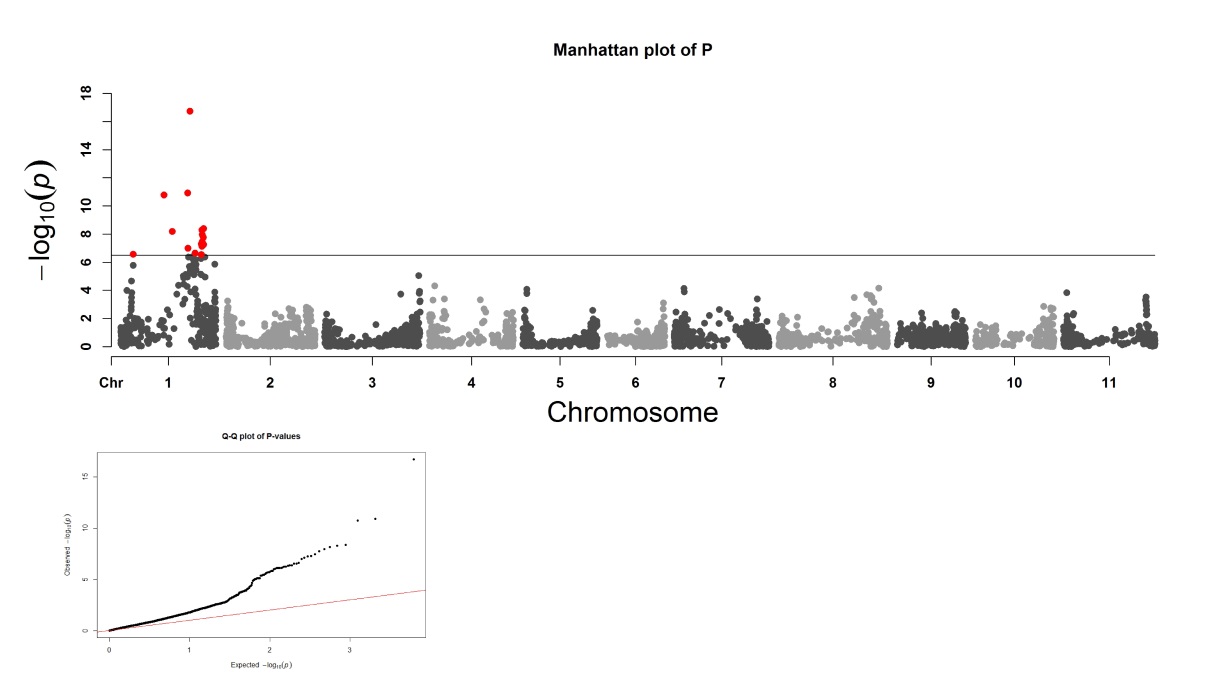
**

1. GLM

**
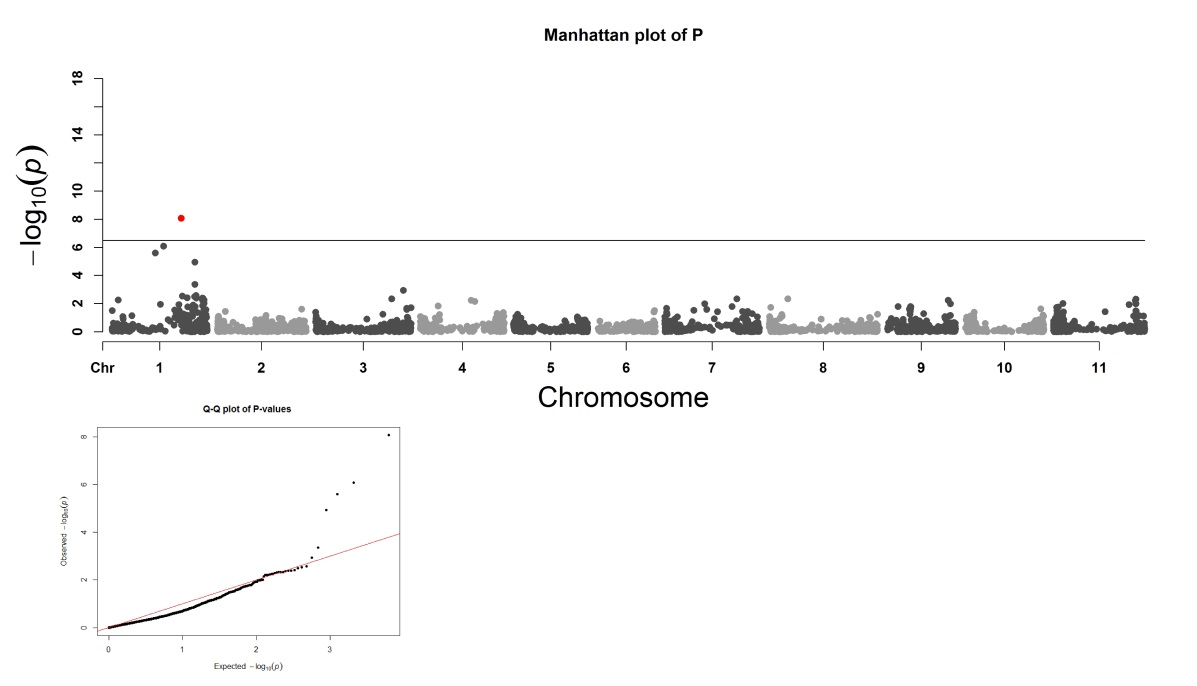
**

1. MLM
