# Supplementary material for: Genetic Diversity, Population Structure, and Linkage Disequilibrium in a Spanish Common Bean Diversity Panel Revealed through Genotyping-by-Sequencing
Source: Genes (Basel). 2018 Oct 23;9(11):518. doi: 10.3390/genes9110518 (PMC6266623; doi:10.3390/genes9110518)
Supplement: Supplementary file 1 [file genes-09-00518-s001.zip › Table_S1_R2.docx]

**Table S1.** **List of accessions included in the Spanish Diversity Panel**. Accessions from the Spanish Core Collection (SCC), including the update SCC, are indicated.

| Panel accession | GP (*Q*)^a^ | Type of material^b^ | Use as snap | Other characteristics |
| --- | --- | --- | --- | --- |
| A25 (Andecha) | AN (0.7) | Old cultivar |  |  |
| A252 | MA (1.0) | Breeding line |  | Source of resistance to anthracnose |
| A321 | MA (1.0) | Breeding line |  | Source of resistance to anthracnose |
| A493 | MA (0.7) | Breeding line |  | Source of resistance to anthracnose |
| AB136 | MA (1.0) | Landrace |  | Anthracnose diferential cultivar |
| Amarilla Capitano | AN (0.9) | Elite cultivar | Yes |  |
| Amethyst | AN (0.7) | Elite cultivar | Yes |  |
| Anellino di Trento | AN (1.0) | Elite cultivar | Yes |  |
| AO-1012-29-3-3A | AN (0.8) | Breeding line |  | Source of resistance to weevil |
| Astrel | AN (0.6) | Elite cultivar | Yes |  |
| BAT93 | MA (1.0) | Breeding line |  | sequenced and annotated genotype |
| Beurre de Roquencourt | AN (0.9) | Old cultivar | Yes |  |
| BGE000993 | MA (1.0) | Landrace (SCC) | Yes |  |
| BGE001144 | MA (1.0) | Landrace (SCC) |  |  |
| BGE001452 | AN (0.8) | Landrace (SCC) | Yes |  |
| BGE001472 | MA (1.0) | Landrace (SCC) |  |  |
| BGE001539 | AN (0.8) | Landrace (SCC) |  |  |
| BGE002016 | AN (1.0) | Landrace (SCC) |  |  |
| BGE002108 | MA (0.9) | Landrace (SCC) |  |  |
| BGE002116 | AN (1.0) | Landrace (SCC) |  |  |
| BGE002132 | AN (0.9) | Landrace (SCC) | Yes |  |
| BGE002134 | AN (1.0) | Landrace (SCC) |  |  |
| BGE002152 | AN (1.0) | Landrace (SCC) |  |  |
| BGE002188 | AN (1.0) | Landrace (SCC) |  |  |
| BGE002189 | MA (1.0) | Landrace (SCC) |  |  |
| BGE002196 | AN (0.9) | Landrace (SCC) |  |  |
| BGE002201 | AN (1.0) | Landrace (SCC) |  |  |
| BGE002204 | AN (0.6) | Landrace (SCC) | Yes |  |
| BGE002207 | AN (1.0) | Landrace (SCC) |  |  |
| BGE002209 | MA (1.0) | Landrace (SCC) |  |  |
| BGE003029 | AN (1.0) | Landrace (SCC) |  |  |
| BGE003037 | MA (1.0) | Landrace (SCC) |  |  |
| BGE003043 | AN (1.0) | Landrace (SCC) | Yes |  |
| BGE003074 | MA (1.0) | Landrace (SCC) |  |  |
| BGE003079 | AN (1.0) | Landrace (SCC) |  |  |
| BGE003121 | AN (0.9) | Landrace (SCC) | Yes |  |
| BGE003122 | AN (1.0) | Landrace (SCC) |  |  |
| BGE003128 | AN (1.0) | Landrace (SCC) |  |  |
| BGE003138 | AN (0.9) | Landrace (SCC) |  |  |
| BGE003139 | MA (1.0) | Landrace (SCC) | Yes |  |
| BGE003161 | MA (1.0) | Landrace (SCC) |  |  |
| BGE003164 | AN (1.0) | Landrace (SCC) |  |  |
| BGE003165 | MA (1.0) | Landrace (SCC) |  |  |
| Panel accession | GP (*Q*)^a^ | Type of material^a^ | Use as snap | Other characteristics |
| BGE003168 | AN (0.9) | Landrace (SCC) |  |  |
| BGE003203 | AN (1.0) | Landrace (SCC) |  |  |
| BGE003246 | AN (1.0) | Landrace (SCC) |  |  |
| BGE003254 | AN (1.0) | Landrace (SCC) |  |  |
| BGE003261 | AN (1.0) | Landrace (SCC) |  |  |
| BGE003274 | AN (0.9) | Landrace (SCC) |  |  |
| BGE003283 | AN (1.0) | Landrace (SCC) | Yes |  |
| BGE003293 | AN (1.0) | Landrace (SCC) |  |  |
| BGE003298 | MA (1.0) | Landrace (SCC) |  |  |
| BGE003404 | AN (1.0) | Landrace (SCC) |  |  |
| BGE003482 | AN (1.0) | Landrace (SCC) |  |  |
| BGE003483 | MA (1.0) | Landrace (SCC) |  |  |
| BGE003487 | MA (1.0) | Landrace (SCC) |  |  |
| BGE003550 | AN (0.9) | Landrace (SCC) |  |  |
| BGE003554 | AN (1.0) | Landrace (SCC) |  |  |
| BGE003555 | AN (1.0) | Landrace (SCC) |  |  |
| BGE003559 | AN (1.0) | Landrace (SCC) |  |  |
| BGE003562 | AN (1.0) | Landrace (SCC) | Yes |  |
| BGE003568 | AN (1.0) | Landrace (SCC) |  |  |
| BGE003626 | AN (1.0) | Landrace (SCC) |  |  |
| BGE003645 | AN (1.0) | Landrace (SCC) | Yes |  |
| BGE003654 | AN (1.0) | Landrace (SCC) | Yes |  |
| BGE003693 | AN (1.0) | Landrace (SCC) |  |  |
| BGE003705 | AN (0.9) | Landrace (SCC) |  |  |
| BGE003746 | AN (1.0) | Landrace (SCC) |  |  |
| BGE003955 | MA (1.0) | Landrace (SCC) |  |  |
| BGE003966 | AN (1.0) | Landrace (SCC) |  |  |
| BGE003997 | AN (0.5) | Landrace (SCC) | Yes |  |
| BGE004000 | AN (1.0) | Landrace (SCC) |  |  |
| BGE004005 | AN (1.0) | Landrace (SCC) |  |  |
| BGE004010 | AN (1.0) | Landrace (SCC) | Yes |  |
| BGE004025 | AN (1.0) | Landrace (SCC) |  |  |
| BGE004034 | AN (1.0) | Landrace (SCC) |  |  |
| BGE004432 | AN (1.0) | Landrace (SCC) |  |  |
| BGE004434 | AN (1.0) | Landrace (SCC) |  |  |
| BGE004435 | AN (0.6) | Landrace (SCC) |  |  |
| BGE004445 | AN (1.0) | Landrace (SCC) | Yes |  |
| BGE004452 | MA (0.9) | Landrace (SCC) |  |  |
| BGE004453 | MA (0.8) | Landrace (SCC) |  |  |
| BGE004454 | MA (1.0) | Landrace (SCC) |  |  |
| BGE004459 | AN (1.0) | Landrace (SCC) |  |  |
| BGE004489 | AN (1.0) | Landrace (SCC) | Yes |  |
| BGE004673 | AN (1.0) | Landrace (SCC) | Yes |  |
| BGE004813 | AN (1.0) | Landrace (SCC) |  |  |
| BGE005439 | AN (0.9) | Landrace (SCC) | Yes |  |
| BGE005475 | MA (1.0) | Landrace (SCC) |  |  |
| BGE005484 | MA (1.0) | Landrace (SCC) |  |  |
| Panel accession | GP (*Q*)^a^ | Type of material^a^ | Use as snap | Other characteristics |
| BGE005487 | AN (1.0) | Landrace (SCC) |  |  |
| BGE008274 | AN (1.0) | Landrace (SCC) |  |  |
| BGE009979 | AN (1.0) | Landrace (SCC) |  |  |
| BGE010387 | AN (0.9) | Landrace (SCC) |  |  |
| BGE010548 | MA (1.0) | Landrace (SCC) |  |  |
| BGE010549 | MA (1.0) | Landrace (SCC) |  |  |
| BGE011016 | AN (0.7) | Landrace (SCC) |  |  |
| BGE011021 | MA (1.0) | Landrace (SCC) |  |  |
| BGE011023 | AN (1.0) | Landrace (SCC) |  |  |
| BGE011026 | AN (1.0) | Landrace (SCC) |  |  |
| BGE011030 | AN (0.9) | Landrace (SCC) |  |  |
| BGE011037 | AN (1.0) | Landrace (SCC) |  |  |
| BGE011058 | AN (0.9) | Landrace (SCC) |  |  |
| BGE011060 | AN (1.0) | Landrace (SCC) |  |  |
| BGE011731 | AN (1.0) | Landrace (SCC) | Yes |  |
| BGE011735 | AN (0.5) | Landrace (SCC) | Yes |  |
| BGE011736 | AN (0.8) | Landrace (SCC) | Yes |  |
| BGE011758 | MA (1.0) | Landrace (SCC) | Yes |  |
| BGE011762 | AN (0.9) | Landrace (SCC) | Yes |  |
| BGE013953 | AN (1.0) | Landrace (SCC) |  |  |
| BGE013962 | AN (0.9) | Landrace (SCC) |  |  |
| BGE013964 | AN (1.0) | Landrace (SCC) | Yes |  |
| BGE013965 | MA (1.0) | Landrace (SCC) |  |  |
| BGE013980 | AN (1.0) | Landrace (SCC) | Yes |  |
| BGE013981 | AN (1.0) | Landrace (SCC) | Yes |  |
| BGE019991 | MA (1.0) | Landrace (SCC) |  |  |
| BGE020003 | AN (0.9) | Landrace (SCC) | Yes |  |
| BGE020030 | AN (1.0) | Landrace (SCC) |  |  |
| BGE020048 | AN (1.0) | Landrace (SCC) |  |  |
| BGE020119 | AN (1.0) | Landrace (SCC) |  |  |
| BGE022070 | AN (0.9) | Landrace (SCC) |  |  |
| BGE022106 | AN (1.0) | Landrace (SCC) |  |  |
| BGE022120 | AN (1.0) | Landrace (SCC) |  |  |
| BGE022129 | AN (0.7) | Landrace (SCC) |  |  |
| BGE022366 | AN (1.0) | Landrace (SCC) |  |  |
| BGE022378 | AN (1.0) | Landrace (SCC) |  |  |
| BGE022494 | AN (0.6) | Landrace (SCC) | Yes |  |
| BGE022504 | AN (0.9) | Landrace (SCC) |  |  |
| BGE022508 | MA (1.0) | Landrace (SCC) |  |  |
| BGE022510 | MA (1.0) | Landrace (SCC) |  |  |
| BGE022512 | MA (1.0) | Landrace (SCC) |  |  |
| BGE022519 | AN (1.0) | Landrace (SCC) |  |  |
| BGE022827 | AN (1.0) | Landrace (SCC) |  |  |
| BGE022831 | MA (1.0) | Landrace (SCC) |  |  |
| BGE022832 | AN (1.0) | Landrace (SCC) |  |  |
| BGE022836 | AN (1.0) | Landrace (SCC) |  |  |
|  |  |  |  |  |
|  |  |  |  |  |
| Panel accession | GP (*Q*)^a^ | Type of material^a^ | Use as snap | Other characteristics |
| BGE022837 | AN (1.0) | Landrace (SCC) | Yes |  |
| BGE023190 | AN (1.0) | Landrace (SCC) | Yes |  |
| BGE023679 | AN (1.0) | Landrace (SCC) |  |  |
| BGE024024 | AN (0.6) | Landrace (SCC) |  |  |
| BGE024038 | AN (1.0) | Landrace (SCC) |  |  |
| BGE024699 | AN (1.0) | Landrace (SCC) |  |  |
| BGE025069 | AN (1.0) | Landrace (SCC) | Yes |  |
| BGE025085 | AN (1.0) | Landrace (SCC) | Yes |  |
| BGE025130 | AN (1.0) | Landrace (SCC) | Yes |  |
| BGE025142 | AN (1.0) | Landrace (SCC) | Yes |  |
| BGE025180 | AN (1.0) | Landrace (SCC) | Yes |  |
| BGE025330 | MA (1.0) | Landrace (SCC) |  |  |
| BGE025739 | AN (1.0) | Landrace (SCC) |  |  |
| BGE025740 | MA (1.0) | Landrace (SCC) |  |  |
| BGE025745 | AN (1.0) | Landrace (SCC) |  |  |
| BGE026146 | AN (1.0) | Landrace (SCC) | Yes |  |
| BGE026151 | AN (1.0) | Landrace (SCC) | Yes |  |
| BGE026155 | AN (1.0) | Landrace (SCC) |  |  |
| BGE026158 | AN (1.0) | Landrace (SCC) |  |  |
| BGE026163 | AN (0.9) | Landrace (SCC) | Yes |  |
| BGE026166 | AN (1.0) | Landrace (SCC) | Yes |  |
| BGE026169 | AN (1.0) | Landrace (SCC) | Yes |  |
| BGE026172 | AN (0.7) | Landrace (SCC) | Yes |  |
| BGE026173 | AN (1.0) | Landrace (SCC) |  |  |
| BGE026186 | MA (1.0) | Landrace (SCC) |  |  |
| BGE026196 | AN (0.9) | Landrace (SCC) |  |  |
| BGE026211 | AN (1.0) | Landrace (SCC) |  |  |
| BGE026222 | AN (1.0) | Landrace (SCC) |  |  |
| BGE027076 | MA (1.0) | Landrace (SCC) |  |  |
| BGE027085 | AN (1.0) | Landrace (SCC) | Yes |  |
| BGE027962 | AN (0.6) | Landrace (SCC) |  |  |
| BGE028939 | AN (0.6) | Landrace (SCC) |  |  |
| BGE028940 | AN (1.0) | Landrace (SCC) | Yes |  |
| BGE028947 | AN (0.6) | Landrace (SCC) |  |  |
| BGE028953 | MA (1.0) | Landrace (SCC) |  |  |
| BGE028958 | MA (1.0) | Landrace (SCC) |  |  |
| BGE028960 | MA (1.0) | Landrace (SCC) | Yes |  |
| BGE028964 | AN (1.0) | Landrace (SCC) |  |  |
| BGE029568 | AN (1.0) | Landrace (SCC) |  |  |
| BGE029569 | AN (1.0) | Landrace (SCC) | Yes |  |
| BGE029581 | AN (1.0) | Landrace (SCC) |  |  |
| BGE029604 | AN (1.0) | Landrace (SCC) |  |  |
| BGE029629 | AN (1.0) | Landrace (SCC) | Yes |  |
| BGE029705 | AN (1.0) | Landrace (SCC) | Yes |  |
| BGE030143 | AN (1.0) | Landrace (SCC) |  |  |
| BGE030453 | AN (1.0) | Landrace (SCC) | Yes |  |
|  |  |  |  |  |
| Panel accession | GP (*Q*)^a^ | Type of material^a^ | Use as snap | Other characteristics |
| BGE030893 | AN (0.9) | Landrace (SCC) |  |  |
| BGE033598 | AN (1.0) | Landrace (SCC) |  |  |
| BGE034306 | AN (1.0) | Landrace (SCC) |  |  |
| BGE036014 | AN (1.0) | Landrace (SCC) |  |  |
| BGE036492 | AN (1.0) | Landrace (SCC) |  |  |
| BGE039249 | MA (1.0) | Landrace (SCC) |  |  |
| BGE039974 | AN (1.0) | Landrace (SCC) |  |  |
| BGE039982 | AN (0.9) | Landrace (SCC) | Yes |  |
| BGE040418 | AN (1.0) | Landrace (SCC) |  |  |
| BGE040514 | AN (1.0) | Landrace (SCC) | S? |  |
| BGE040527 | AN (1.0) | Landrace (SCC) | Yes |  |
| BGE043356 | MA (0.8) | Landrace (update SCC) |  |  |
| BGE043359 | AN (0.9) | Landrace (update SCC) |  |  |
| BGE043364 | AN (0.9) | Landrace (update SCC) |  |  |
| BGE043365 | MA (1.0) | Landrace (update SCC) | Yes |  |
| BGE043366 | MA (0.9) | Landrace (update SCC) | Yes |  |
| BGV008281 | AN (1.0) | Landrace (update SCC) | Yes |  |
| BGV008519 | MA (1.0) | Landrace (update SCC) |  |  |
| BGV013606 | MA (1.0) | Landrace (update SCC) |  |  |
| BGV014077 | MA (1.0) | Landrace (update SCC) |  |  |
| BGV014083 | AN (0.9) | Landrace (update SCC) |  |  |
| Bilma | MA (0.9) | Elite cultivar | Yes |  |
| Bina | AN (0.7) | Elite cultivar | Yes |  |
| Blauhilde | MA (0.9) | Elite cultivar | Yes |  |
| Bluevetta | AN (0.6) | Elite cultivar | Yes |  |
| Boca de Dragón | AN (0.9) | Elite cultivar | Yes |  |
| Borlotto Rosso | AN (1.0) | Elite cultivar | Yes |  |
| BRB130 | AN (0.8) | Breeding line |  | Source of resistance to potyvirus |
| Brown Dutch | AN (0.6) | Elite cultivar | Yes |  |
| Buenos Aires Roja | AN (1.0) | Old cultivar | Yes |  |
| CN_220 | MA (1.0) | Landrace (update SCC) |  |  |
| CN_222 | MA (1.0) | Landrace (update SCC) |  |  |
| CN_223 | MA (0.9) | Landrace (update SCC) |  |  |
| CN_225 | AN (1.0) | Landrace (update SCC) |  |  |
| CN_226 | AN (0.9) | Landrace (update SCC) |  |  |
| CN_227 | AN (0.9) | Landrace (update SCC) |  |  |
| CN_228 | MA (1.0) | Landrace (update SCC) |  |  |
| CN_230 | MA (1.0) | Landrace (update SCC) |  |  |
| CN_231 | AN (1.0) | Landrace (update SCC) |  |  |
| CN_241 | AN (1.0) | Landrace (update SCC) |  |  |
| CN_250 | MA (0.9) | Landrace (update SCC) |  |  |
| Cobra | AN (0.6) | Elite cultivar | Yes |  |
| Contender | AN (1.0) | Old cultivar | Yes |  |
| Cornell49242 | MA (1.0) | Breeding line |  | Anthracnose diferential cultivar |
| Donna | MA (0.9) | Elite cultivar | Yes |  |
| DOR364 | MA (1.0) | Breeding line |  |  |
|  |  |  |  |  |
| Panel accession | GP (*Q*)^a^ | Type of material^a^ | Use as snap | Other characteristics |
| Dorabel | AN (0.6) | Elite cultivar | Yes |  |
| Dublette | AN (0.9) | Elite cultivar | Yes |  |
| Duplika | AN (0.9) | Elite cultivar | Yes |  |
| Emilia | AN (0.7) | Elite cultivar | Yes |  |
| Fin de Bagnols | AN (0.8) | Old cultivar | Yes |  |
| Finbel | AN (0.8) | Elite cultivar | Yes |  |
| Florencia | MA (0.9) | Elite cultivar | Yes |  |
| G12508 | MA (1.0) | Landrace |  |  |
| G12910 | MA (1.0) | Landrace |  |  |
| G13467 | MA (0.9) | Landrace |  |  |
| G19833 | AN (0.9) | Landrace |  | Sequenced and annotated genotype |
| G02333 | MA (0.9) | Landrace |  | Anthracnose diferential cultivar |
| Garonel | AN (0.8) | Elite cultivar | Yes |  |
| Garrafal enana | AN (1.0) | Old cultivar | Yes | Traditional Spanish snap cultivar |
| Garrafal Oro | AN (1.0) | Old cultivar | Yes | Traditional Spanish snap cultivar |
| Gloire de Saumur | AN (0.9) | Old cultivar | Yes |  |
| Golden Teepee | AN (0.9) | Elite cultivar | Yes |  |
| Goldmarie | MA (0.9) | Elite cultivar | Yes |  |
| Helda | MA (1.0) | Old cultivar | Yes | Traditional Spanish snap cultivar |
| Ilerda | AN (0.9) | Elite cultivar | Yes |  |
| IVT7214 | MA (1.0) | Breeding line |  | Source of resistance to potyvirus |
| Kaboon | AN (0.9) | Old cultivar |  | Source of resistance to anthracnose |
| Le Victoire | AN (0.9) | Old cultivar | Yes |  |
| Manteca de los Mercados | AN (0.9) | Elite cultivar | Yes |  |
| Manteca Rocquencourt | AN (1.0) | Elite cultivar | Yes |  |
| Maravilla de Venecia | AN (1.0) | Old cultivar | Yes |  |
| Marbel | AN (0.7) | Elite cultivar | Yes |  |
| Marconi | MA (0.9) | Elite cultivar | Yes |  |
| MDRK | AN (1.0) | Old cultivar |  | Anthracnose diferential cultivar |
| Meraviglia_di_Venezia | AN (0.9) | Elite cultivar | Yes |  |
| Mex222 | MA (0.7) | Old cultivar | Yes | Anthracnose diferential cultivar |
| Michelite | MA (1.0) | Old cultivar |  | Anthracnose diferential cultivar |
| Midas | AN (0.9) | Elite cultivar | Yes |  |
| Monel | AN (0.8) | Elite cultivar | Yes |  |
| Musica | MA (0.9) | Elite cultivar | Yes |  |
| N11277 | MA (1.0) | Landrace |  |  |
| N11283 | MA (1.0) | Landrace |  |  |
| Nassau | AN (0.9) | Elite cultivar | Yes |  |
| Neckargold | MA (0.8) | Elite cultivar | Yes |  |
| Novirex | AN (0.9) | Elite cultivar | Yes |  |
| Nuria | AN (0.9) | Elite cultivar | Yes |  |
| Oxinel | AN (0.8) | Elite cultivar | Yes |  |
| Patxi | MA (0.9) | Elite cultivar | Yes |  |
| Perfeccion Negra Polo | AN (0.7) | Elite cultivar | Yes |  |
| PerryMarrow | AN (1.0) | Old cultivar |  | Anthracnose diferential cultivar |
|  |  |  |  |  |
| Panel accession | GP (*Q*)^a^ | Type of material^a^ | Use as snap | Other characteristics |
| PI207262 | MA (1.0) | Landrace |  | Anthracnose diferential cultivar |
| Pinzessa | AN (0.6) | Elite cultivar | Yes |  |
| Planeta | MA (1.0) | Elite cultivar | Yes |  |
| Porrillo Sintetico | MA (1.0) | Old cultivar |  | Source of resistance to powdery mildew |
| PR1464-4 | MA (0.9) | Breeding line |  | Source of resistance to weevil |
| PR1464-6 | MA (0.9) | Breeding line |  | Source of resistance to weevil |
| Primel | AN (0.9) | Elite cultivar | Yes |  |
| Rocdor | AN (0.9) | Elite cultivar | Yes |  |
| Roma II | AN (1.0) | Elite cultivar | Yes |  |
| Royalnel | AN (0.7) | Elite cultivar | Yes |  |
| Sacha | MA (0.9) | Elite cultivar | Yes |  |
| Sanilac | MA (1.0) | Old cultivar |  |  |
| SanilacBc6_Are | MA (1.0) | Breeding line |  | Source of resistance to anthracnose |
| Saxa | AN (0.9) | Elite cultivar | Yes |  |
| SEL1308 | MA (1.0) | Breeding line |  | Source of resistance to anthracnose |
| SEL1360 | MA (1.0) | Breeding line |  | Source of resistance to anthracnose |
| Slenderette | AN (0.8) | Elite cultivar | Yes |  |
| Superba | AN (0.9) | Elite cultivar | Yes |  |
| Tendergreen | AN (1.0) | Old cultivar | Yes | *Pseudomonas* diferential cultivar |
| TO | MA (0.9) | Old cultivar |  | Anthracnose diferential cultivar |
| Triomphe de Farcy | AN (0.8) | Old cultivar | Yes |  |
| TU | MA (0.7) | Old cultivar |  | Anthracnose diferential cultivar |
| V169 | AN (0.7) | Landrace |  |  |
| V203 | AN (1.0) | Landrace |  |  |
| V205 | AN (1.0) | Landrace |  |  |
| V206 | AN (1.0) | Landrace |  |  |
| V207 | AN (1.0) | Landrace | Yes |  |
| V208 | AN (0.6) | Landrace |  |  |
| V213 | AN (1.0) | Landrace |  |  |
| V226 | MA (1.0) | Landrace |  |  |
| V381 | MA (1.0) | Landrace | Yes |  |
| Vitalis | MA (0.9) | Elite cultivar | Yes |  |
| Widusa | MA (0.9) | Old cultivar | Yes | Anthracnose diferential cultivar |
| X2776 | AN (0.8) | Elite cultivar |  |  |
| Xana | AN (0.8) | Elite cultivar |  |  |

^a^ GP, gene pool and *Q* value assigned in the structure analysis. AN, Andean. MA, Mesoamerican

^b^Old cultivar, cultivars that have been commercialized or described many years ago
