# Supplementary material for: Genetic Diversity, Population Structure, and Linkage Disequilibrium in a Spanish Common Bean Diversity Panel Revealed through Genotyping-by-Sequencing
Source: Genes (Basel). 2018 Oct 23;9(11):518. doi: 10.3390/genes9110518 (PMC6266623; doi:10.3390/genes9110518)
Supplement: Supplementary file 1 [file genes-09-00518-s001.zip › Table_S2_R2.docx]

**Table S2.** **Distribution of the 3,099 SNPs**. Distribution per chromosome of the 3099 SNPs used in this work. Except for minimum distance, values are expressed in Mega base pair (Mb).

|  |  |  | SNPs |  |  |
| --- | --- | --- | --- | --- | --- |
| Chr | Size | Number | Average dist | Min dist | max dist |
| 1 | 52.20 | 291 | 0.18 | 745 | 2.99 |
| 2 | 49.08 | 399 | 0.12 | 898 | 1.46 |
| 3 | 52.31 | 359 | 0.13 | 532 | 2.80 |
| 4 | 44.50 | 199 | 0.23 | 501 | 3.51 |
| 5 | 40.82 | 222 | 0.18 | 597 | 3.30 |
| 6 | 31.98 | 238 | 0.13 | 590 | 1.38 |
| 7 | 51.76 | 349 | 0.15 | 514 | 2.70 |
| 8 | 59.66 | 317 | 0.19 | 609 | 3.05 |
| 9 | 37.47 | 307 | 0.12 | 616 | 1.81 |
| 10 | 43.30 | 179 | 0.24 | 550 | 3.99 |
| 11 | 50.38 | 239 | 0.21 | 681 | 3.63 |
| *Average* |  | *282* | *0.17* | *621* | *2.79* |
