# Supplementary material for: Genetic Diversity, Population Structure, and Linkage Disequilibrium in a Spanish Common Bean Diversity Panel Revealed through Genotyping-by-Sequencing
Source: Genes (Basel). 2018 Oct 23;9(11):518. doi: 10.3390/genes9110518 (PMC6266623; doi:10.3390/genes9110518)
Supplement: Supplementary file 1 [file genes-09-00518-s001.zip › Table_S3_R2.docx]

**Table S3. Tag sequences**. Tag sequences (strand +) containing the 15 SNP that showed the greatest contribution to the differentiation between gene pools

| Name | Contrib | Tag sequence | Gene | Localization |
| --- | --- | --- | --- | --- |
| s9_13978576 | 0.0628 | TGTGGCCTGGCCAA[T/C]GTAGTTTCAAGCTGAACCGGGACTGAGATTTTGGGAAGCTGGATGGCTG | PHAVU_009G089900g | 3`UTR |
| s7_5756712 | 0.0624 | CAGCAAGAACAGCATTAGATATCTACAGCATTGATA[A/T]ACTAATGTATCAGTATAACAACATCAA | PHAVU_007G065000g | Intron |
| s7_5831598 | 0.0624 | CAGCGGAATGAGGCAGGGTTACCTGGGATCGTAGTTGATCAATCTGCTCCATATACA[G/A]TTGAGC | PHAVU_007G065500g | Intron |
| s9_13780397 | 0.0617 | GAATTTATTTGAAATTAGAGAGATGAGAGGAGAGAAAAAAT[G/T]ACCATGGCGAGGACATGCGCTG | PHAVU_009G088400g | Exon |
| s1_6033703 | 0.0613 | TCCTTGTTAAGGTGCATGGGAGG[T/C]AATTCTTTCTCCACAACTAAGGAATAGTGACCTTCAGCTG | PHAVU_001G052900g | Exon |
| s8_4499339 | 0.0611 | CTGC[A/C]TTCCCACTCTTGTGCATTCCATTATATACAATTCCTGCACCTGCAGAGGCTTCTACTTC | PHAVU_008G051100g | Exon |
| s8_4345326 | 0.0610 | CTGCATAGATGCCG[T/A]TAAAGGTCTTACACAAAAAAAGTTATTGTCACCATTTTCTATTTGCAGT | PHAVU_008G049500g/  PHAVU_008G049600g | intergenic |
| s1_6197061 | 0.0609 | CAGCAAGAAGCAGAAAAAGGTTTGATTCCTGAA[C/T]TCGTAAAGGTCATAAAAGAAACAAGGGAAC | PHAVU_001G053900g | Exon |
| s7_5887308 | 0.0608 | TTTGTGTAAGGAGAACC[T/A]CCCCTTTCAAGGACTAAAACTCTTGCACCTTGCGAGAGAGTTGCTG | PHAVU_007G066200g | Exon |
| s9_11995387 | 0.0607 | CTGCTTCGGAAGCGGCGGAGGCGGATCGTTGAACGTCGTAGACGGCTAC[C/T]GAGGCATCGGAGGC | PHAVU_009G071700g | Exon |
| s4_43950011 | 0.0606 | CTGCACAATACCACCT[T/A]GCTCTTACTTTTAGACAGCTCTCGCAGGCTCGTGCAGTTCTAGCCCC | PHAVU_004G157300g | Exon |
| s8_4866955 | 0.0606 | CAGCCGG[C/T]CGGCTTGTGGCGATCCTGAGCATCTGCCTCACAATCTATGGGATCTCATCCTTCAA | PHAVU_008G054400g | Exon |
| s9_20452212 | 0.0606 | CAGCTTGAGATTATGGATGA[C/T]GGATATAAATGGAGGAAGTACGGAAAGAAGACAGTGAAGAACA | PHAVU_009G138900g | Exon |
| s7_4755652 | 0.0605 | CAGCGTCCGCTCGTACGTCGCCCTTCCCC[T/C]CGATTACCAGGACTTTGCGCCCCGCCCTCTTGAC | PHAVU_007G057400g | Exon |
| s9_12807237 | 0.0604 | CTGCACTAATGACCCTACACGC[A/T]CTAAAGAAAAGTTAAGCCAATAAAAAACTAATACTATTGAT | PHAVU_009G078900g | Intron |
